# The Florida Harvester Ant, *Pogonomyrmex badius*, Relies on Germination to Consume Large Seeds

**DOI:** 10.1371/journal.pone.0166907

**Published:** 2016-11-28

**Authors:** Walter R. Tschinkel, Christina L. Kwapich

**Affiliations:** 1 Department of Biological Science, Florida State University, Tallahassee, Florida, United States of America; 2 School of Life Sciences, Social Insect Research Group, Arizona State University, Tempe, Arizona, United States of America; University of California San Diego, UNITED STATES

## Abstract

The Florida harvester ant, *Pogonomyrmex badius*, is one of many ant species and genera that stores large numbers of seeds in damp, underground chambers for later consumption. A comparison of the sizes of seeds recovered from storage chambers with those of seed husks discarded following consumption revealed that the used seeds are far smaller than stored seeds. This difference in use-rate was confirmed in field and laboratory colonies by offering marked seeds of various sizes and monitoring the appearance of size-specific chaff. Because foragers collect a range of seed sizes but only open small seeds, large seeds accumulate, forming 70% or more of the weight of seed stores. Major workers increase the rates at which small and medium seeds are opened, but do not increase the size range of opened seeds. Experiments limiting ant access to portions of natural seed chambers showed that seeds germinate during storage, but that the ants rapidly remove them. When offered alongside non germinating seeds, germinating seeds were preferentially fed to larvae. The rate of germination during the annual cycle was determined by both burial in artificial chambers at various depths and under four laboratory temperatures. The germination rate depends upon the species of seed, the soil/laboratory temperature and/or the elapsed time. The seasonal soil temperature cycle generated germination patterns that vary with the mix of locally-available seeds. Taken together, exploitation of germination greatly increases the resources available to the ants in space and time. While the largest seeds may have the nutritional value of 15 small seeds, the inability of workers to open large seeds at will precludes them from rapid use during catastrophic events. The harvester ant’s approach to seed harvesting is therefore two-pronged, with both immediate and delayed payoffs arising from the tendency to forage for a wide variety of seeds sizes.

## Introduction

The exploitation of seeds as food has evolved independently several times among ant species. Roughly 18 genera of ants regularly harvest seeds, often acting as the dominant seed consumers in the grasslands, deserts and forests they inhabit [1]. Most harvester ant species are not specialists, but collect and store a variety of seed types. For example, in North America, *Pogonomyrmex occidentalis* collects up to 45 seed species [2,3] and *Pogonomyrmex owyheei* 29 species [4]. *Veromessor pergandei* collects 29 different seed species from 24 genera in Death Valley alone [5]. Nevertheless, foragers do discriminate among the broader array of species available in their environment. Such seed preferences have been attributed to the body size of the ant [6,7,8], seed abundance [9], caloric value [10,11,12], toxicity [13] and novelty[11].

The degree to which seed harvesting ant species rely on seeds as a singular source of nutrition also varies across genera. Some species, like *Veromessor pergandei*, are almost exclusively granivorous, while species of *Pogonomyrmex* range from a partial dependence on insect protein, fungus, bird feces and other plant material, to an almost exclusively seed diet [14,15,16]. The relative ratio of assimilated seed and insect protein by *Pogonomyrmex badius* larvae differs based on physical caste [17] and season (Kwapich and Tschinkel, unpublished).

### Seed storage

Seeds collected by harvester ants are stored in damp, underground granaries, which, in the case of *P*. *badiu*s, are usually between 40 cm and 100 cm below ground [18]. Total stores in this species may exceed half a kilogram and include more than 300,000 seeds [19]. MacKay [16,20] reported that over the annual cycle, seed storage in *P*. *rugosus* ranged from 10 to 70 mg of seeds per worker, with generally much lower rates in *P*. *subnitidus*, and none in *P*. *montanus*. Large colonies of *P*. *occidentalis* collect approximately 30 seeds per active hour, and house an estimated 58,000 seeds in the nest, 78% of which are from grass species alone [14]. *Pogonomyrmex occidentalis* has been reported not to overwinter with seeds, implying that seeds in some species may be accessed or discarded within the span of a single annual cycle (Willard & Crowell, 1965). However, this is contradicted by Lavigne [3] whose excavations of *P*. *occidentalis* recovered seeds throughout the year. Little is known about the residence time of seeds in ant nests or if particular seed species are used preferentially. The relative ratios of seeds entering and chaff exiting can be monitored, but no attempts have been made to monitor the fate of individual seeds or to estimate relative seed residence times.

It has been suggested that caches act as a surplus to tide the colony over during predation events [20,21] or to prevent starvation when resources are limited. Tevis [22,23] attributed the undiminished success of one *V*. *pergandei* population to seed stores amassed before the onset of a 12 year drought. However, experiments on *P*. *badius* demonstrated that colonies do not access their seed stores when prevented from foraging for 7 to 60 days, at the expense of larval survival, alate production and worker fat stores [13,15]. Likewise, when the larval population of a nest is experimentally doubled, the abundant seeds stores are not accessed and the larvae do not survive [24]. These case studies suggest that seed storage may be more beneficial to long term survival, rather than an immediate buffer against catastrophe.

### Seed germination

To effectively store thousands of seeds in the ground over time, ants must deny them the temperature, light and moisture cycles necessary for germination. *Pheidole providens*, *Pheidole diffusa* and *Solenopsis geminata* do so by drying damp seeds outside of the nest following rain [25,26], while *Messor barbarus* takes a more direct approach by destroying the radicle of intact seeds [27]. The occurrence of seed chambers at habitat-specific depths suggest that below-ground gradients may also play a role in preventing germination [3,18,19]. Finally, the high-density environment of a seed cache could signal seeds to self-inhibit germination to avoid competition with other seeds or seedlings [28].

Despite the apparent strategies to prevent the germination of stored seeds by harvesters, many ant species share mutualistic relationships with plants and actively promote the germination of, and plant numerous myrmecophorous seed species [9,29,30,31]. Only anecdotal accounts of granivorous ants cohabitating with germinating seeds exist, and the relationship is decidedly predatory. In 1879, Treat [32] reported that captive *P*. *badius* workers only processed wild seeds that were already “swollen and sprouted,” and that deep storage chambers were free of germinating seeds while superficial chambers housed both larvae and germinating seeds on warm mornings. Wheeler [33] noted that during winter months, deep seed storage chambers contained masses of sprouting seeds, which were disposed of on the surface. He described these seeds as having sprouted “too far to be fit for food,” suggesting that seeds are not inactivated by *Pogonomyrmex* workers during storage, and that seeds are normally processed shortly after germination during the active season. During the winter months, the absence of larvae, and the concentration of less active adults in the lower-most chambers of the nest may contribute to the accumulation of germinating seeds.

### Seed milling and consumption

Seeds are typically opened by repeated abrasion by the mandibles of workers, as evidenced by the age-related wearing of mandibular teeth in *Pogonomyrmex* [34]. In polymorphic species, large headed workers may even specialize on seed milling, but relative rates of seed cracking with and without major workers has not been reported. Following cracking, Goetsch [35] described the production of “ant bread” by the repeated chewing and addition of saliva by *Messor* workers. Like natural germination, this step may aid in the malting process and accelerate the availability of sugars for hungry larvae.

Ant bread and whole seed particles are consumed by adult workers [14], and distributed in large chunks to larvae. During feeding, each larva curls her head forward to feed on the seed pieces deposited on the upward-facing, ventral surface. In *Veromessor pergandei*, larvae in turn produce a liquid which is shared with adult caregivers via trophallaxis [36]. The same behavior has been observed in the seed harvester *P*. *badius* (Danielle Taylor, unpublished).

*Pogonomyrmex* workers have been observed opening seeds by physical action of the mandibles. However, When *P*. *badius* colonies are prevented from foraging, the seed cache is not depleted, and larvae die of starvation in as little as 7 days [13,15]. One explanation for this finding is that the ants are physically incapable of opening the majority of their cache. While some seeds may be immediately useful to ants, there may be a significant delay between collection and payoff for those of a greater size or toughness. MacGown et al. [37], observed clear differences in the more than 15 species of seeds that appeared in storage chambers and as remnant chaff on the surface of *P*. *badius* nests in Mississippi. Six species were found only in the granaries, 11 only in the middens, and only one in both. Interestingly, many seeds in the upper-most granary chambers were germinating.

### The basis of this study

Past reports have suggested that harvester ants may sometimes exploit germination to make seeds, especially large seeds, usable, but this possibility has long remained largely an open question. After having excavated and censused approximately 200 *P*. *badius* nests over many years, and determining the size distribution of the stored seeds [18,19], we noted that the discarded husks of seeds in the middens were mostly derived from small seeds, while the chambers contained mostly large seeds. A similar observation was also made by McGowan et al. [37]. This raised several questions. If the ants rarely use large seeds, why do they collect and store so many? *P*. *badius* is reputed to have evolved large major workers in order to allow them to open larger seeds effectively [38], but are they actually able to open the large seeds? If not, is the accumulation of large seeds simply an expected but "unintended" consequence of the ants collecting all seeds, but being able to use only the small ones? More generally, do the ants prevent stored seeds from germinating, or do they depend upon their germination? It seems possible that ants could influence germination by moving seeds across vertical gradients of the nest, which may exceed 2 meters in depth [39]. If the ants do rely on germination, the staggered germination of seeds of various ages and types could help explain why they select such a wide variety of seed species.

A preliminary experiment in which seeds taken from *P*. *badius* nests were buried in porous Petri dishes in Dec, 2013, or subjected to several laboratory temperature conditions found that some of the seeds of various sizes germinated quite readily and at substantial frequencies. This opened up the possibility that the ants are actually "farming" the seeds to germinate, rather than preventing them from doing so. We explored these and related questions by means of a number of experiments and observations.

## Materials and Methods

The logical structure of our study is as follows: we begin by showing that most of the seeds the ants use are small, whereas those they store are large; next we show that, indeed, this is best explained by the ants' inability to open large seeds; suspecting that maybe the ants rely on germination to use large seeds, we follow with experiments on the conditions for seed germination; having found that seeds do germinate under conditions approximating those in ant nests, we ask if the ants could use such germinating seeds; having found they do, we show that seeds *actually germinate* in the ants' own storage chambers, and are used quickly. Finally, we quantify the ants' stored seeds with respect to those they can and cannot open, identifying many of them to species.

### Field site

The study population of Florida harvester ant, *P*. *badius*, is located in a 23 ha site (latitude 30.3587, longitude -84.4177) about 16 km southwest of Tallahassee, Florida, USA, within the sandhills ecotype of the Apalachicola National Forest. The site, Ant Heaven, consists of excessively drained sandy soil occupying a slope to a wetland and stream, causing its water table to be depressed (>5 m at the maximum), thereby making it suitable for *P*. *badius* and *Solenopsis geminata*, as well as several drought-resistant species of plants such as *Opuntia* and *Nolina*. The forest consists of longleaf pines (*Pinus palustris*) planted ca. 1975, turkey oak (*Quercus laevis*), bluejack oak (*Quercus incana*), occasional sand pines (*Pinus clausa*) and sand live oak (*Quercus geminata*). Because the soil had been disturbed in the early 1970s, the natural ground cover of wiregrass (*Aristada stricta*) is absent, replaced by broomsedge (*Andropogon* spp.) and several other successional species of grasses, herbs and shrubs. The same disturbance may have helped establish this dense population of *P*. *badius*, whose nests are easily spotted because the ants decorate the excavated sediment disc with a layer of charcoal bits (mostly the ends of burned pine needles). The black charcoal contrasts sharply with the light-colored sand or litter.

This project was carried out under US Forest Service, Apalachicola National Forest permit number APA56302, Expiration Date: 12/31/2017. *Pogonomyrmex badius* is not a protected species.

### Seeds

Seeds were collected from field colonies of *P*. *badius* by excavation, using the methods of Tschinkel [18,39] in which chambers are exposed horizontally and emptied, one by one. The ants store seeds in large numbers in chambers mostly located 30 to 80 cm below ground [39]. The seeds usually took up most of the chamber area and were piled several layers deep with just enough head space for ants to maneuver. For this study, we used three batches of seeds, the first collected in 1989 from 31 colonies [18] (hereafter, the 1989 seeds), the second from four colonies in November 2014 (the 2014 seeds), and the third from nine colonies in September 2015 (the 2015 seeds). The collected seeds were air-dried in the laboratory and separated by means of U.S. Standard Testing Sieves Nos. 8, 10, 12, 14, 16, 18, 20, 25, 30, 35. The range of dimensions of the seeds thus separated are shown in Table 1, along with the mean weight, s.d. and relative weight (relative to the smallest seeds) of each size class. To the extent possible, we identified seeds to species using Landers and Johnson [40] and Rosene and Freeman [41], as well as the seed collection at the University of Florida Herbarium in Gainesville, and the Robert Godfrey Herbarium at Florida State University in Tallahassee. We will publish these identifications along with images elsewhere, under the title, "An illustrated guide to seeds found in nests of the Florida harvester ant, *Pogonomyrmex badius"*, authored by Tschinkel and Dominguez.

**Table 1:** Seed sizes. The range of dimensions and mean weight of seeds in the 1989 and 2014 samples captured on each of the U.S. Standard Testing Sieves. Sieve openings were given on the sieves. The 1989 samples were from the studies by Tschinkel [19,42]. Data for the 1989 seeds can be found in S1 Table.

Sieve Size	Size range, mm (= sieve opening)	1989 Mean Seed Weight, mg	S.D. of 1989 seeds, mg	1989 Relative weight	2014 size classes, Mean Seed Weight, mg	S.D. of 2014 seeds
8+	>2.36	14.45	6.89	42.5		
10	2.00–2.36	7.30	1.96	21.5	10.9*	4.24
12	1.7–2.0	5.28	1.94	15.5		
14	1.4–1.7	2.31	0.76	6.79	2.61	0.31
16	1.18–1.4	1.47	0.34	4.32		
18	1.00–1.18	0.83	0.16	2.44	1.49**	0.19
20	0.85–1.0	0.63	0.28	1.85	0.65	0.24
25	0.71–0.85	0.51	0.33	1.50		
30	0.60–0.71	0.37	0.26	1.08		
35	0.50–0.60	0.34	0.26	1.00		

* Included larger seeds as well.

** Included size 16 seeds as well

Most of the experiments were run using the 2014 seeds, separated into four size classes (Sieves No. 12+, 14, 18, 20). Each of these classes was dominated by the seeds of one or two species, but three also contained other species (described in Results below). For this reason, we will refer to these groups by size rather than species.

### Seed marking

In several experiments, seeds of different sizes or different experimental treatments were marked by spraying with 10% fluorescent printers' ink (Gans Ink and Supply Co.) in ether. Available colors fluoresced orange, yellow, green and blue under ultraviolet light. Seeds or seed husks were recovered under UV light from the middens of field colonies, the nest chambers, or the nests and arenas of laboratory colonies.

### Seed use in relation to seed size

When the ants have access to a range of seed sizes, the frequency of discarded husks on the middens is a record of the rate at which these sizes are processed and used. Colonies were offered 30 seeds of each of four sizes (No. 12+, 14, 18 and 20) each size having been sprayed with a different color of fluorescent printers' ink. Five replicates were run on laboratory colonies and nine on field colonies. In the laboratory colonies, a UV light was used daily to search for discarded husks and intact seeds in the laboratory arena and nest, and the location and condition of each fluorescent item was recorded. In the field colonies, a portable darkroom was placed over the nest disc every two to three days and a UV light was used to search the nest disc and midden for fluorescent items (all of which were later checked in the laboratory).

### Seed storage in relation to seed size and use

The husks of seeds that the ants have opened and used are discarded on the middens, and serve as a record of the seeds the ants have eaten. Seed use and seed stores were assessed in three different ways. Seed chaff was collected from six colonies and compared to the contents of seed chambers following the excavation of 12 nests. For a different set of colonies, seed size distribution was compared to seeds taken from laden foragers returning to their nests (n = 6 colonies; mean 140 seeds per colony, s.d. 78) to reveal the sizes actually collected by foragers. Finally, an estimate of the seeds contained in the colony's stores was generated by robbing seeds from trail workers for several hours during colony relocations (n = 7 colonies), an event during which the colony moves all stored seeds to the new nest [43].Seed size distributions were compared to the size distribution of husks in the middens, and in the case of the seeds robbed during relocations, to several samples taken from the newly constructed nests for a number of days. The old chaff represented the sizes of seeds actually used over the time the colony was in residence (mean of about one year), and the new chaff represented the sizes of seeds currently being used.

### Worker size and seed use

Major workers have often been assumed or shown to function as seed millers, allowing colonies to increase the size of seeds they are able to open [44,45]. To further explore this idea, six unrelated colony fragments were set up with either minor workers only (mean 450 +/-315 s.d.), or the same number of minor workers plus majors (mean 27; range 9–68). The mean minor/major ratio was 20+/- 10 (s.d.; range 13–35)). All nests were given a mean of 30 larvae (range 25–35) and 14 pupae (range 5–23), and housed without their queen in plaster-bottom Petri dish nests in foraging arenas with water and sugar water available *ad libitum*. Each nest was offered 30 seeds of each of 4 sizes (No. 12, 14, 18, 20), and each size was marked by spraying with a different color of fluorescent printers ink. Nests were monitored for one week (two were monitored for three weeks, n = 6), the number of each seed size in the arena and in the nest were monitored once daily or more. Husks of seeds that had been opened were recovered from the trash pile.

### Burial experiment

To investigate the natural germination rates of seeds stored at different depths below ground under physical conditions similar to those in ant nests, seeds were monitored for germination at 4 depths that either fell within or outside of the natural depth of seed storage chambers in *P*. *badius* nests. Seeds were collected from *P*. *badius* nests in November 2014 and again in September 2015 for comparative purposes. The experiment was begun in January 28, 2015. Seeds were sifted into size classes using the U.S. Standard Testing Sieves, and four sizes were set aside for burial, those on sieves number 12, 14, 18 and 20 (Table 1). A circle 60 mm in diameter was cut out of the bottoms of plastic 100 mm Petri dishes, and the dishes were filled with a 5 to 6 mm layer of a 20% orthodontal plaster, 80% sand mixture and allowed to set. The plaster bottoms were divided into four equal sectors with aluminum strips. Each of the four sectors received one of the sizes of seeds according to these approximate numbers: 70 No. 12; 100 No. 14; 50 No. 18 and 25 No. 20. A total of 20 such dishes was prepared for each run of the experiment so that 5 replicates could be simultaneously run at four different depths of burial.

The dishes were buried at Ant Heaven in the Apalachicola National Forest (approx. latitude 30.35852, longitude -84.41746) by using a soil corer to make five holes 160 mm in diameter and 80 cm deep. Dishes with seeds were buried at 80 cm, 40 cm, 15 cm and 5 cm and left for one month. In natural nests, seeds are stored below 30cm. The exposed plaster bottoms assured that soil moisture equilibrated with the moisture in the dishes. One of the five replicates was provided with an i-button Thermochron (Maxim Integrated, San Jose, California) to record the temperature at each depth at hourly intervals for the month of burial. The dishes were recovered after one month. Beginning January 28, 2015, a fresh set of five replicates was buried every two months, for a total of six sets (Table 2). Beginning in September 2015, an additional set of five replicates was added using the seeds collected in September 2015, so that the remaining runs included seeds collected in November 2014 and September 2015. Prior to use, all seeds were stored dry at laboratory temperatures (approx. 24°C).

**Table 2:** Seed burial experiment. Dates of seed burial and recovery, as well as source of seeds used. All dates except the last are 2015. Each of the six runs was composed of five replicates at four depths using four sizes of seeds (12+, 14, 18, 20).

Run Number	Source of seeds	Month of burial
1	Nov. 2014	February
2	Nov. 2014	April
3	Nov. 2014	June
4	Nov. 2014	August
5	Nov. 2014, Sep. 2015	October
6	Nov. 2014, Sep. 2015	December
7*	Nov. 2014, w/o No. 20*	February 2016*

* This replicate had missing cells, and was excluded from ANOVA.

The seeds in the recovered dishes were evaluated for germination under a dissecting microscope in the laboratory. Data for the burial experiment can be found in S2 Table.

### Laboratory germination tests

Samples from the same sets of seeds used for the burial experiment were also subjected to germination tests at four different temperatures (10°, 15°, 24° and 32°C) in the laboratory. Petri dishes were prepared as for burial, but the bottom was left intact, and the plaster moistened occasionally, as needed. In contrast to the burial experiment, which of necessity was checked only at the end of the one-month run, the laboratory seeds were checked for germination every two or three days, and germinated seeds counted and removed. These laboratory tests were run for one month in parallel to the field, burial experiment. Data for the laboratory experiment can be found in S3 Table.

### Germinating and non-germinating large seeds

#### Field experiments

Size 14 seeds (1.4 mm) represented the smallest seed size that the ants did not readily open, as determined in laboratory experiments. Size 14 seeds that had been sprayed with orange fluorescent printers' ink were germinated on wet filter paper in the laboratory. Controls were ungerminated seeds of the same size, sprayed with green fluorescent ink. Each of nine field colonies was offered 50 germinating and 50 non-germinating marked seeds. Beginning the next day, a portable dark room was placed over each nest disc and the disc and midden checked for marked seeds or seed husks under UV light. Each colony’s midden was checked every few days for 15 to 43 days. All fluorescent items were collected for verification in the laboratory. Because husks are only half of the seed coat, the number of recovered husks was divided by two to yield the number of seeds these husks represented.

#### Laboratory experiments

**Fig 1:**
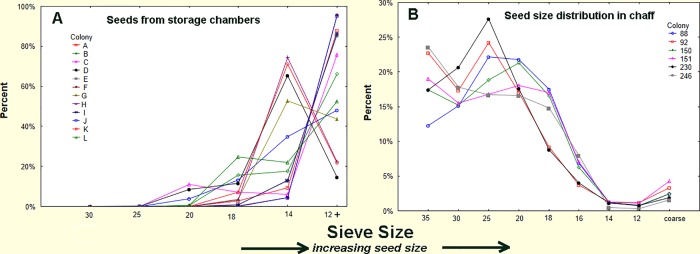
Size distribution of seeds and husks. The size of seeds stored (A) and consumed (B) by *P*. *badius*. Size distribution of consumed seeds was determined from the chaff discarded on the nest disc by the ants, while that of stored seeds was determined directly by excavating nests and collecting their seeds. Stored seeds are mostly large, whereas consumed seeds are mostly small. Each line represents a colony.

Size 14 seeds were marked and germinated as in the field experiment above. Germinated seeds were dyed in 0.5% rhodamine B for about two days, producing deeply dyed and strongly fluorescent cotyledons and embryos (Fig 1A, 1B and 1C). In order to produce dyed control seeds stained with a different color, 0.1 mm holes were drilled in the marked non-germinated control seeds, and the seeds soaked in 0.5% methylene blue for two days (Fig 1D). External dye was rinsed off both types of seeds with water, and the seeds dried. The holes in the non-germinating seeds were sealed with green fluorescent printers' ink and allowed to harden for two days. Equal numbers of marked, dyed germinated and non-germinated seeds were then offered to laboratory colonies. Small colonies received five of each, and larger colonies with more larvae received ten of each. Colonies were monitored twice a day for 7 to 10 days, and the number of larvae that were pink or blue was recorded. Pink larvae were checked under UV light, fluorescing intensely. In one replicate, the dye colors of germinated and non-germinated seeds were reversed.

### Data analysis

Data were subjected to analysis of variance (ANOVA) or Chi-square test, as appropriate, in Statistica 12. In most experiments, colony was the replicate. Treatments included seed size, germination/non-germination, major/minor workers, and seed access/no access. For ANOVAs of the burial and laboratory temperature experiments, the fraction germinated was arcsin-square-root transformed to stabilize the variance. In December 2015, the last of the 2014 size 20 seeds were used, so that the burial and laboratory germination tests for February 2016 were run without these seeds. As this created missing cells, most ANOVAs were run without these February results. As the differences between analyses with and without the February 2016 data were small, we report only the results that excluded the February 2016 sample.

## Results

### Basic observations: seed use in relation to seed size

Several experiments and observations showed that the sizes of seeds that the ants use (as revealed by the discarded seed husks in their chaff) are much smaller than the sizes that they store or collect. Fig 1 reveals that the size distribution of husks recently deposited in middens, and seeds recovered from excavated underground chambers were always very different in their size distribution. Although these data were taken from two different sets of colonies, it is clear that the seeds the ants are able to use are mostly size 18 (1mm diameter) and smaller, while those stored in chambers are mostly larger than size 18.

**Fig 2:**
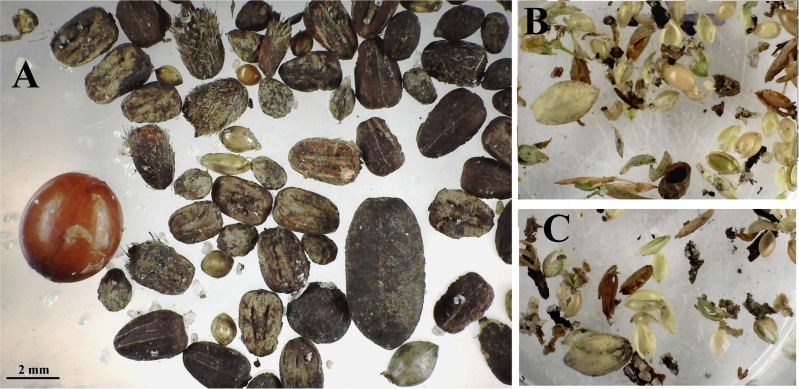
Representative seed and chaff samples. (A) Representative samples of seeds taken from seed-carrying workers during colony relocations; (B and C) chaff taken from middens before or after move completion, respectively. The chaff represents seeds opened and used by the ants, whereas the moved seeds represent the stores in underground chambers.

**Fig 3:**
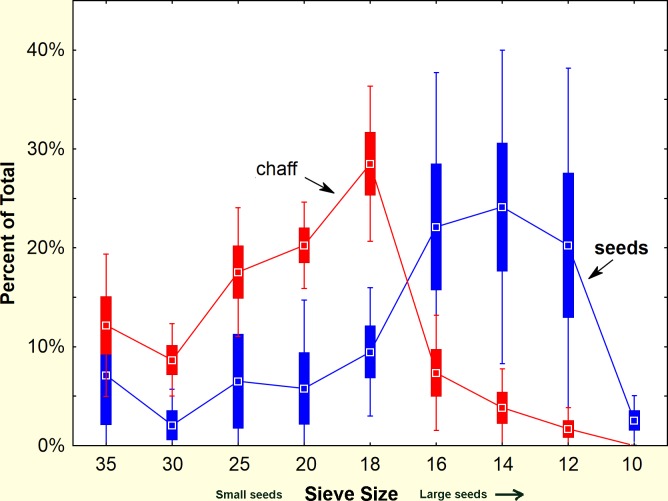
Comparison of the sizes of stored and used seeds. The size distribution of seeds taken from seed-transporting workers during nest relocation, and seeds taken from the chaff. (n = 7 colonies). The chaff represents seeds opened and used by the ants, whereas the moved seeds represent the stores in underground chambers. Bars represent SE and whiskers 95% confidence intervals.

A more direct and non-destructive within-colony comparison of stored and used seed size distribution was made by robbing seed-carrying workers during nest relocations. During such relocations, the ants move all of the stored seeds from their old nest to the new one [43],carrying them on distinct trails between the nests. The seeds borne by workers on such trails represent the underground stores, whereas husks taken from the chaff at the old nest revealed the average use over an extended period, and the chaff at the new nest revealed recent use over a few days. Representative samples of seeds and chaff are shown in Fig 2. Fig 3 shows that seeds larger than size 18 predominate in the storage chambers, whereas seeds of size 18 and smaller make up most of the chaff. Seed use after the move is very similar to before. Thus, large seeds accumulate while small seeds are used more rapidly.

**Fig 4:**
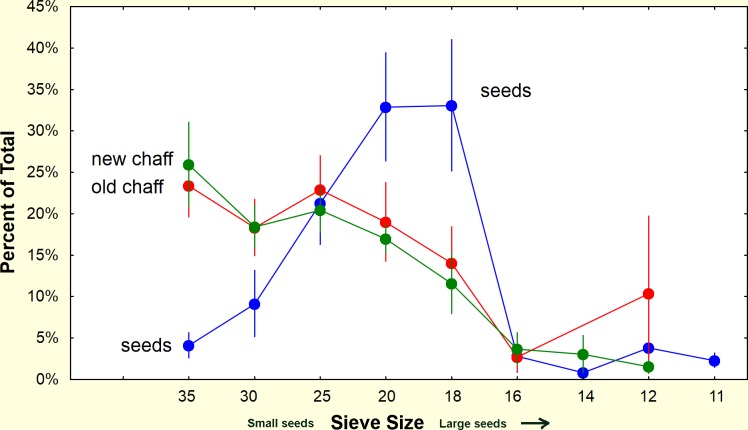
Foraged seed size comparison. The size distribution of seeds taken from returning foragers, and seeds taken from old and new chaff. The chaff represents seeds opened and used by the ants, and consists of seeds that are generally smaller than those brought in by foragers. (n = 6 colonies; representing 21 to 242 seeds per colony and 66 to 1011 chaff pieces per colony. Whiskers = 95% confidence intervals

These observations suggest a comparison of the sizes of seeds that foragers bring into the colony with the seeds the ants actually use. Laden foragers returning to the nest of six different colonies were therefore robbed of their seeds (mean 140 +/-78 s.d. seeds per colony), and the distribution of these seed sizes compared to the sizes of the recently-deposited and old chaff (both divided by 2) in these same colonies (mean 360 +/-350 s.d. seed-equivalents, per colony). Fig 4 shows that foragers collected more larger seeds than represented in either the old or new chaff. Although size 18 was most frequently collected, its husks made up only a small part of the chaff. A few large seeds (size 12, 14) were also taken from the burdened ants, seeds that we will show below, cannot be opened by the ants. Thus, the ants not only store more large seeds than small, they also collect more large seeds. Conversely, they use more small seeds and collect fewer of them.

### Seed size experiments: can the ants open large seeds?

The disparity between the sizes of stored seeds and used seeds suggested that the ants cannot open large seeds. The composition of the seed stores are the product of two size-specific rates, the rate of input of each size and its rate of use. Given that overall seed collection rates exceed use rates, if the rate of use of smaller seeds exceeds that of larger seeds, then larger seeds will accumulate relative to the smaller. It seemed likely that the ants are able to crack open the small seeds (as evidenced by the discarded husks), but not the large ones, causing the large seeds gradually to accumulate. This accumulation can conceivably go on for the entire life of the colony, because the colony moves its entire seed store every time it relocates [43,46].

#### Laboratory

**Fig 5:**
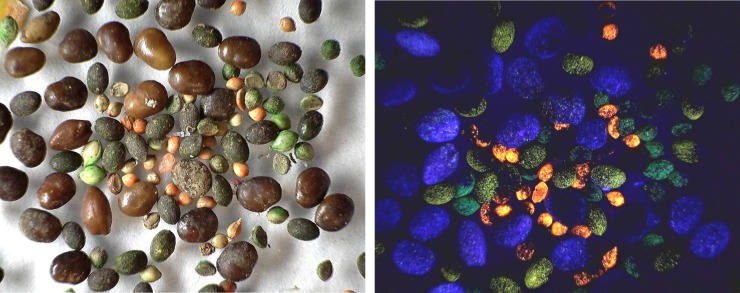
Seeds of four sizes marked with fluorescent ink. A. Under visible light; B. Under ultraviolet light. Small = orange; medium = green; large = yellow; very large = blue.

The ability of the ants to open seeds of a range of sizes was tested by offering five laboratory colonies seeds of four sizes (No. 12 (very large), 14 (large), 18 (medium), 20(small)), each sprayed a different color with fluorescent printers' ink (Fig 5). Thirty seeds of each color were offered simultaneously in the arena of laboratory colonies. The location and disposition of the seeds was monitored for 2 weeks. Successful opening of seeds was indicated by finding the marked husks in the trash pile. Size could be assigned from color, even if the husk had been broken into pieces.

**Fig 6:**
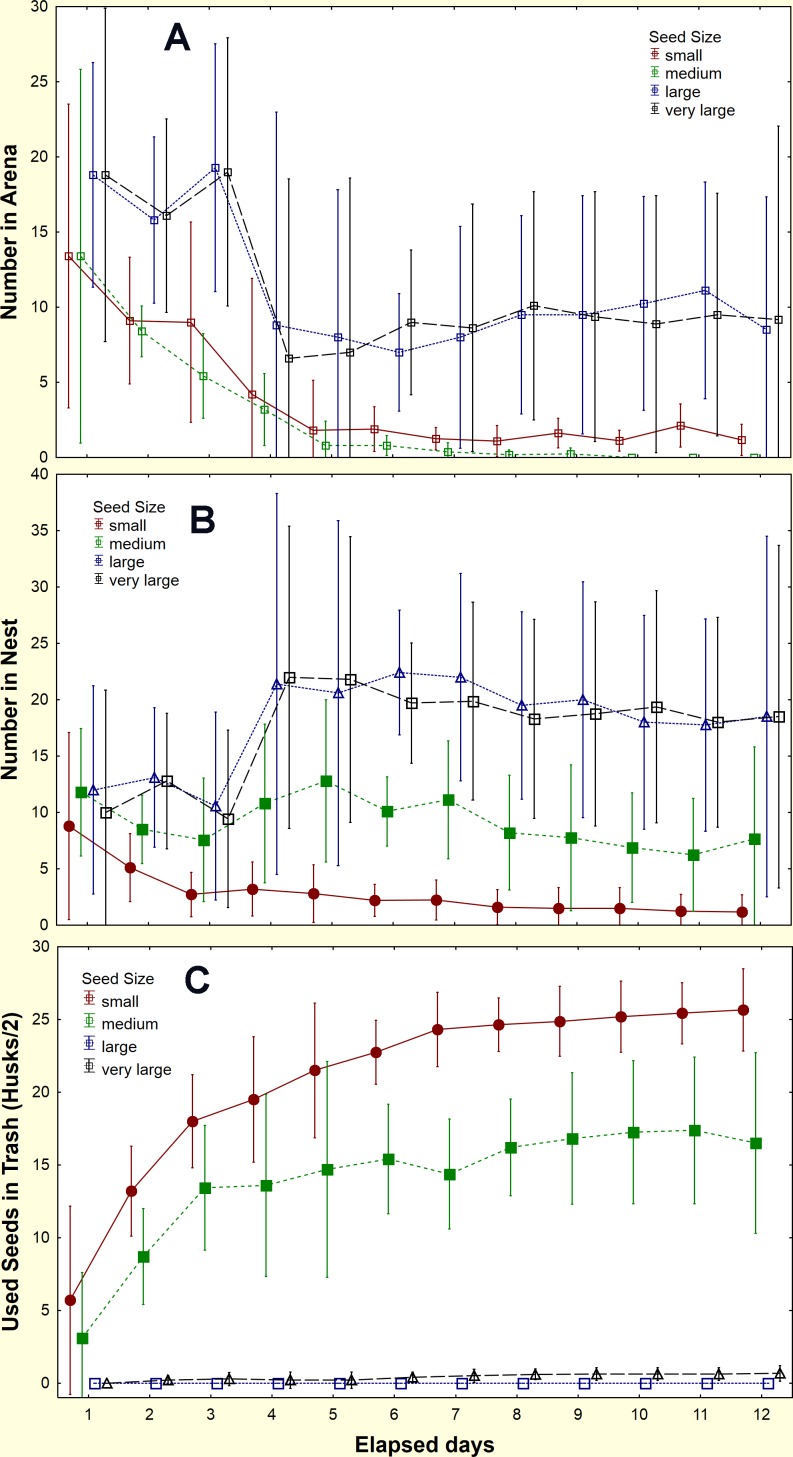
The location and disposition of four sizes of seeds in laboratory colonies. Large and very large seeds were never opened and eaten, but were retrieved from the arena (top panel) and stored in the nest (middle panel). By contrast, small seeds, and to a lesser extent, medium seeds were rapidly husked, eaten and the husks discarded in the trash pile (bottom panel). Seed were retained on sieves as follows: small, No. 20; medium, No. 18, large, No. 14, very large, No. 12. Error bars = 95% confidence interval (n = 5 colonies)

In laboratory colonies, 80% of the husks of small seeds and 60% of those of medium seeds appeared as chaff in the trash pile within about six days, indicating that they had been opened and probably eaten (Fig 6C). In contrast, almost none of the large and very large seeds were opened even after 12 days (Fig 6A and 6B). All sizes of seeds were brought into the nests, although this was more rapid for the two small sizes than the large (Fig 6A and 6B).

**Fig 7:**
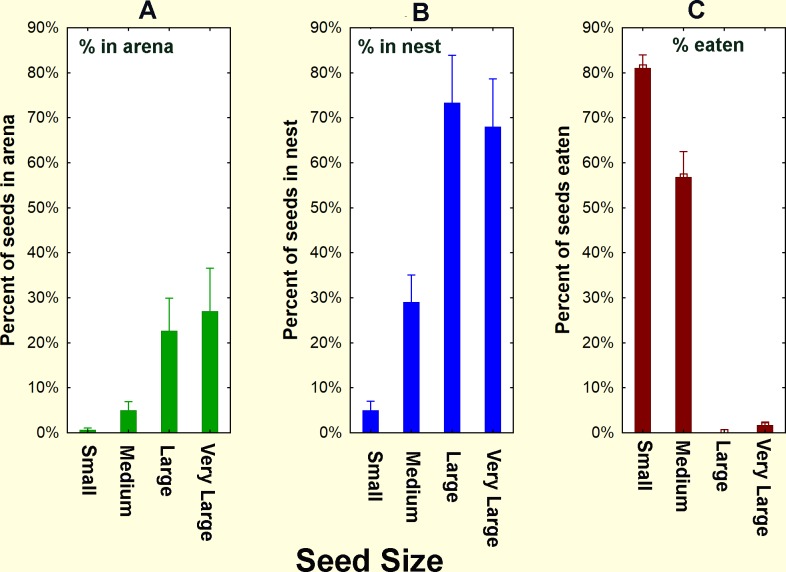
Final disposition of seeds in relation to seed size, as percent of the 30 offered seeds of each size. A. and B. Large and very large seeds were retrieved from the arena and stored in the nest but were almost never opened and eaten, C. By contrast, small seeds, and to a lesser extent, medium seeds were rapidly husked, eaten and the husks discarded in the trash pile. N = 5 colonies. Seeds were retained on sieves as follows: small, No. 20; medium, No. 18, large, No. 14, very large, No. 12. Error bars = 95% confidence interval. ANOVA for seed size: in arena, F_3,16_ = 1.39; n.s.; unprocessed in nest, F_3,16_ = 6.57; p< 0.005; eaten, F_3,16_ = 100.5; p< 0.00001

By the end of the experiment, few seeds of any size remained in the arena (Fig 7A; seed size, F_3,16_ = 1.39; n.s.). Because the small sizes were opened and their remains discarded, whereas the large sizes were not, the larger seeds accumulated in the nest, so that about 70% of the larger seeds were to be found, unprocessed, in the nest (Fig 7B; seed size, F_3,16_ = 6.57; p< 0.005), while 82% and 58% of the small and medium seeds had been eaten (Fig 7C; seed size, F_3,16_ = 100.5; p< 0.00001). Most of this movement and use occurred within six days or less, so that the proportions of each were similar between day six and day 12 (ANOVA; F_3,30_ = 2.43; n.s.).

#### Field

Do field colonies, like laboratory colonies, also use seeds preferentially by size? In the field, 30 seeds of each color were offered simultaneously just off the nest disc (n = 9 colonies), and the location and disposition of the seeds and seed husks was monitored for two weeks. The pattern of appearance of marked husks in the middens followed a pattern similar to that in laboratory colonies. Whereas all nine colonies retrieved all 30 of each of the four seed sizes, only the husks of small and medium seeds appeared on the middens. A mean per colony of 17.3 (s.d. 8.43) seed equivalents (husks/2) were recovered for small seeds, 10.0 (s.d. 4.62) for medium seeds, 0.6 (s.d. 0.77) for large seeds and none for very large seeds (one-way ANOVA F_3,32_ = 26.4; p< 0.00001). The number of opened small seeds was significantly greater than medium seeds (Tukey's HSD test, p< 0.005). The number of whole seeds discarded in the middens was low for all sizes: 0.6, 0.7, 1.9, and 0, in order of increasing size.

Thus, the field experiments verify the laboratory findings—the ants are able to open only the small and medium seeds, discarding their husks on the middens. Moreover, even among seeds the ants can open, the rate of opening declines with seed size.

### Role of major workers in processing seeds

The literature suggests that major workers play a positive role in seed milling, especially of large seeds, and this was tested in a laboratory experiment. The laboratory experiment above was repeated except that each experimental colony was divided into a nest with major workers and one without (mean 450 minor workers, range 111–900; mean 28 major workers, range 9–68), each receiving a mean of 30 larvae (range 25–35) and 14 pupae (range 5–23). Both types of nest were queenless, as the queen, a few workers and a few larvae had been set aside for later reunification. All nests were offered 30 size-specifically marked seeds of each of the four sizes, and the experiment was run for 7 days (two were run for 19 days), noting the location and condition of the seeds, as above. Six replicates were completed.

**Fig 8:**
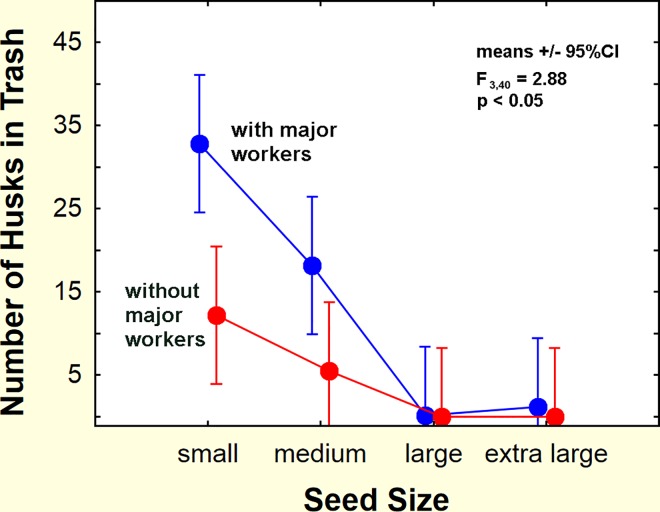
Seeds opened with and without majors present. When majors were present in the nests, more seeds were opened, but the maximum size opened was the same as in nests lacking majors (n = 6 colonies).

The presence of major workers increased the number of small and medium seeds processed significantly. When majors were present a mean of 33 husks of small seeds, and 18 medium seeds were collected from the trash. When they were absent, 12 small and 5.5 medium seeds were collected (Fig 8). Husks of large and extra large seeds were almost absent (mean <1). Thus, the ants almost exclusively milled small and medium seeds, whether majors were present or not, but when majors were present, they milled significantly more small and medium seeds (Chi-square comparison within experimental colonies: small seeds Х^2^ = 39, p< 0.000001; medium seeds Х^2^ = 27, p< 0.00005; large seeds Х^2^ = 0.50, n.s.; extra large seeds Х^2^ = 3.5, n.s.). In all treatments together, more small than medium seeds were processed (mean 23 vs. 12), suggesting that ant size limits the ability of workers to open seeds, a conclusion strengthened by the fact that almost no large and very large seeds were opened. In contrast, the mean number of seeds present in the nest did not differ with either seed size or worker size, averaging between 13 and 18 seeds (this number varied with time as well as changing when seeds were processed) (two-way ANOVA: n.s.). The mean number of seeds remaining (uncollected) in the arena was higher when majors were absent (18 vs. 9.3) (ANOVA: major/minor—F_1,40_ = 12.0; p <0.002), suggesting that either majors actively collected more seeds or stimulated minors to do so. The collection of seeds of all sizes, combined with the inability to open seeds larger than size 18 (even by majors) leads to the accumulation of large seeds in the underground stores.

### Germinating seeds are used preferentially

In view of the inability of the ants to open large seeds, it seemed possible that they are able to use germinating seeds, especially when these are too large for them to open. In germinating seeds, the husk splits open, revealing the embryo, cotyledons and radicle, all easily masticated tissues, and enzymes convert starch to sugars, making digestion by animals easier. To test whether the ants rely on germination for access to large seeds, nine field colonies were each simultaneously offered two groups of 50 size-14 seeds (large, a size they normally cannot open), one group germinating and husk-marked with one color of fluorescent ink, and the other non-germinating and marked with another color.

**Fig 9:**
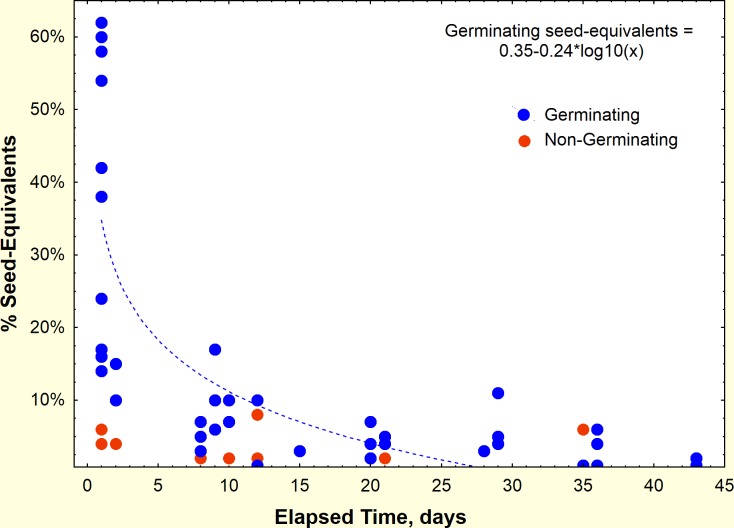
The appearance of marked seed husks in the middens of field colonies that had been offered marked germinating and non-germinating seeds. Germinating seeds (blue symbols) were far more likely to appear in the middens than non-germinated (red symbols). Because husks were mostly only half of the seed coat, the number was divided by two to estimate whole seed-equivalents.

The husks of the germinated seeds were far more likely to be recovered from the middens. Most of the husks of germinating seeds appeared quickly with 40% appearing within a day. After a week, the rate was mostly less than 10% (Fig 9). Summed over the duration of the experiment, 74% of germinated seed-equivalents (husks/2) appeared in the middens, but only 2% of non-germinated seeds did so (95% CI, 0–5.0%; one-way ANOVA: F_1,16_ = 512, p< 0.000001). No germinated seeds were recovered whole from the midden, while about 16% of non-germinated seeds were (95% CI, 2–30%; F_1,16_ = 7.27, p< 0.02). The total recovery of seeds or seed equivalents was thus 74% for germinated, and 18% for non-germinated (but most were unopened). Altogether, the total number of seeds recovered over the entire period was much higher for germinating than non-germinating seeds (one-way ANOVA: F_1,16_ = 69.3, p< 0.000001). Clearly, the ants were able to open the germinated seeds at a much higher rate than the non-germinated, discarding the husks. The unopened, non-germinated seeds were presumably stored in seed chambers. This process was next studied in laboratory experiments in which the fate of the seed interior could be directly observed.

### Germinating seeds are preferentially fed to larvae

#### Laboratory

**Fig 10:**
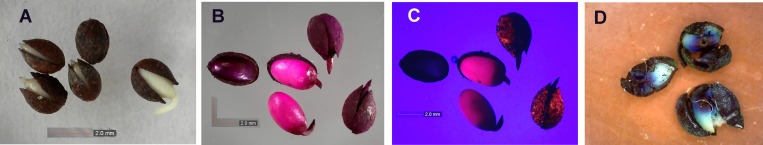
Marked and dyed No. 14 seeds. (A) Germinating seeds; (B) dyed and marked germinating seeds; (C) dyed and marked germinating seeds under UV light. Note fluorescence of both the husk and the seed interior; and (D) drilled and dyed non-germinating seeds, broken open to reveal the blue-dyed tissue inside.

**Fig 11:**
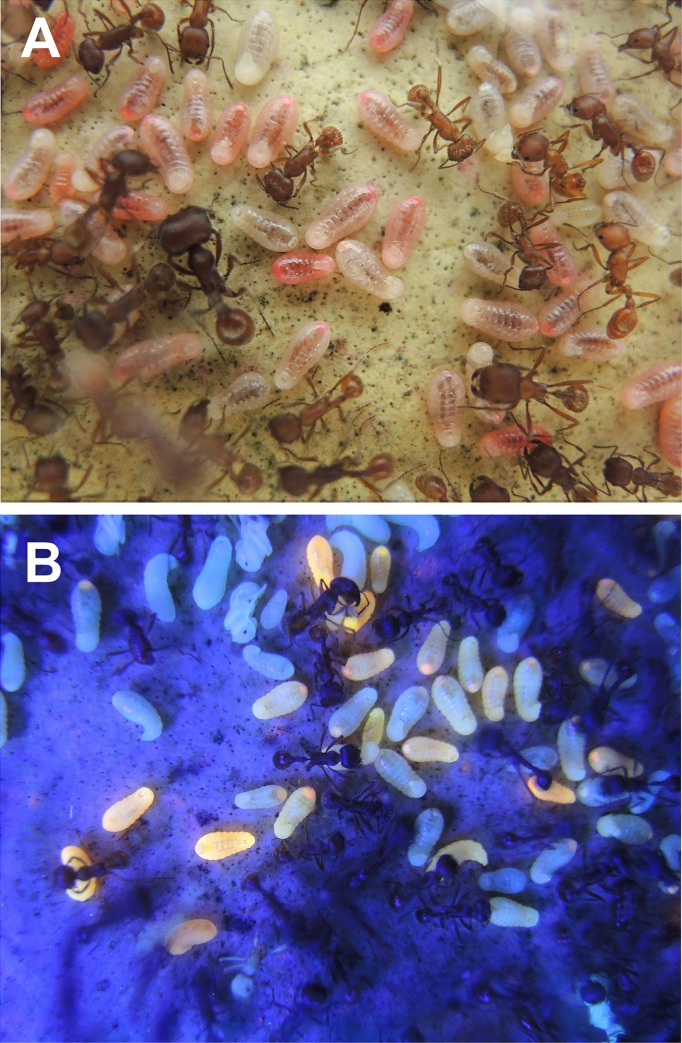
The results of the dyed germinating and non-germinating seed experiment. Dyed germinating seeds were fed to larvae in the laboratory, causing them to appear pink (A) and to fluoresce under UV light (B). From the midgut, the rhodamine dye is translocated into the rectum, appearing as pink and fluorescent dots at the posterior end of the larvae, like the light on a caboose.

Having shown above that the ants quickly use the germinating (but not the non-germinating) seeds, it remained to test whether germinating seeds were actually fed to larvae. This was done by offering colonies germinating seeds whose cotyledons had been dyed with rhodamine and whose husk had been color-marked with fluorescent ink. Controls were color marked, but non-germinating seeds. Husk-marked and rhodamine-dyed germinating seeds (Fig 10) were rapidly collected, the husk removed and the cotyledons fed to larvae, so that within two days, a mean of 45% of larvae were visibly pink and fluoresced brightly under UV light (Fig 11). The dye appeared in the midguts, but was soon translocated to the rectum. Many larvae had chunks of pink-dyed seed on their venters, and appeared to masticate these chunks. Almost all of the marked husks of germinated seeds appeared in the trash piles within four days or less. Marked non-germinating seeds were taken into the nest where an average of 60% of them remained, unopened, for the duration of the experiment, although a few larvae with blue in the rectum appeared after a week, suggesting that the ants were occasionally able to open intact, non-germinating seeds. However, this occurred in only three of the five experimental nests.

#### Field

Were germinating seeds similarly fed to larvae in field colonies as they were in the laboratory colonies above? The laboratory experiment above was repeated on field colonies by offering 25 marked and dyed germinating and 25 non-germinating seeds, and excavating the colony two days later to assess the proportion of brood that were pink and/or fluoresced. A mean of 173 (s.d. 51) brood were recovered from the nests and assessed for having ingested dyed seed material. Fluorescence was more sensitive than pink coloration—54% of the fluorescing immatures were also visibly pink. We also recovered marked seeds and husks.

Two to three days after being offered dyed germinating No. 14 seeds, 24% of larvae and 12% of pharate pupae, or 17% of total brood (s.d. 16%; n = 8) had received dye. Ten percent (s.d. 10%) contained enough dye to appear visibly pink. As pharate pupae do not feed, these had to have fed and transformed in the previous two or three days. *Because the ants had their own large seed stores available to them*, *this rapid dyeing of brood shows that the ants preferentially feed germinating seeds to their larvae*. The husks of these dyed seeds were found in the trash in five of the eight colonies, with a mean of 17 (s.d. 15) husks per colony. No marked, non-germinating seeds were found in any of the trash piles, but were recovered from the seed stores.

### Conditions for germination

The above experiments and observations show that the ants are able to process and consume large seeds after they germinate, but do these large seeds actually germinate at a high enough rate to provide the ants with significant nutrition? For these experiments, seeds were placed on damp plaster in Petri dishes without ants. One set of dishes was buried at several depths at Ant Heaven while the other was kept at several laboratory temperatures. Burial subjected the seeds to physical conditions similar to those prevailing in ant nests.

#### Burial experiment, 2015

**Fig 12:**
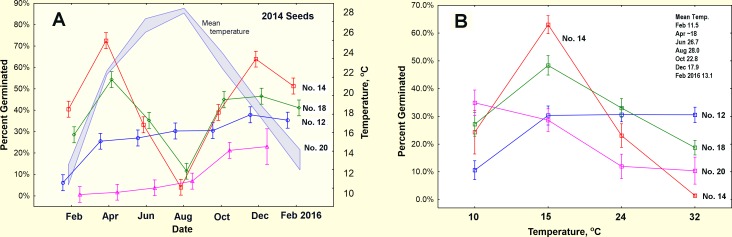
Results of the seed burial and laboratory germination experiments. (A) Fraction of seeds germinating in the burial experiment, and (B) in the parallel laboratory temperature experiment. (A). Burial date and seed size together with their interactions accounted for 93% of the factor variance. Burial depth and its interactions accounted for only 6% of the factor variance, and are not shown in this graph. The similarity of the effects of laboratory temperature and burial date suggests that soil temperature is largely responsible for stimulating/inhibiting germination in the burial experiment. Mean soil temperature is shown as a thick, pale blue line in panel A with the scale on the right axis. (B). Incubation temperature and seed size together with their interactions accounted for 89% of the variance in germination rate. Run number and its interactions explained less than 11% of the factor variance and are not shown in this graph. Bars = 95% CI.

The percent of seeds that germinated in the burial experiment showed strong patterns by date of burial (season) and seed size (Fig 12A) but not burial depth. Date, seed size and the date by seed size interaction accounted for 93% of the variance in fraction germinating that was associated with the independent factors (ANOVA of the arcsin-square root of the fraction germinating, S4 Table). For ease of interpretation, the figures show the untransformed percent germination. Seed size had the greatest main effect (F_3,367_ = 267; p<<0.00001) accounting for 35% of the factor-associated variance. Size 14 and 18 seeds had the highest rate of germination, size 12 intermediate and size 20 the lowest (Fig 12, right panel). The April and December runs both had high germination rates, while August had the lowest. However, the effect of date (season) depended on seed size, and this interaction accounted for 32% of the factor-associated variance (interaction of date by seed size: F_9,367_ = 47.8; p<0.00001). Thus, both size 14 and 18 seeds showed a strong maximum in April and December separated by a very low minimum in August. In contrast, both size 12 and 20 seeds showed only a gradual increase in germination throughout the year.

Date of burial had the third largest main effect (F_3,367_ = 119; p<<0.00001), accounting for 26% of the factor-associated variance. Germination peaked in April and December, and was low in August.

Germination rate was not related to depth of burial (F_3,367_ = 2.1; n.s.). Depth's interactions with both date and seed size were significant, but small relative to other factors (date by depth: F_9,367_ = 3.0; p<0.0002; size by depth: F_9,367_ = 2.3; p<0.02), and are not considered further here.

#### Simultaneous laboratory temperature experiment

In the parallel laboratory runs at four constant temperatures (10, 15, 24, 32°C; mean coefficient of temperature variation 1.8%; range 0.7% to 3.8%), both seed size and temperature generated strong patterns of germination (Fig 12B). Temperature, seed size, and the temperature by seed size interaction accounted for 71% of the variance associated with the independent factors, most of which can be seen in the two-way interaction plots in Fig 12B (ANOVA of the arcsine square root transformed fractions, S4 Table). Not surprisingly, temperature had a large main effect (F_3,368_ = 260; p<<0.00001), accounting for 28% of the factor-associated variance. Overall germination rates did not simply increase with temperature—they were moderate at 10°C, peaked at 15°C, dropped at 24°C and were very low at 32°C.

The effect of temperature was different for seeds of different sizes—germination of sizes 14 and 18 peaked strongly at 15°C, dropping off at both higher and lower temperatures, whereas germination of size 12 increased from 10°C to 15°C, but changed little thereafter. Size 20 seeds showed a steady decline in germination over the whole tested range, dropping from 35% at 10°C down to 10% at 32°C. The effect of temperature was only moderately related to run (F_9,368_ = 16.1, p < 0.0001), with only the 10°C treatment showing a decline with run.

Seed size showed both a large main effect (F_3,368_ = 45.9; p<<0.00001; 5% of factor variance) with maximum rate of germination for size 18 seeds, as well as a strong interaction with temperature (F_9,368_ = 118; p<<0.00001; 38% of factor variance) that was driven largely by the strong peak for seed size 14 and 18 at 15°C, the indifferent temperature response of size 12, and the declining rate of germination of size 20 as temperature increased (Fig 12B).

Date of the run (the equivalent of date/season in the burial experiment) and its three interactions had significant effects on germination, but altogether they accounted for less than 19% of the factor-associated variance, contrasting with the much larger effects of temperature, seed size and their interactions (66%). Germination rate differed by date of the run (main effect of date, F_3,368_ = 9.3; p<<0.00001), but date interacted with temperature (F_3,368_ = 16.1; p<<0.00001), with germination rate declining with date of the run at 10°C but not other temperatures. The effect of run also depended on seed size (F_3,368_ = 15.2; p<<0.00001) with mid-sized seed germination declining with run while large and small seed germination changed little. The effect of date of the run can be interpreted as elapsed time, or seed aging, separate from the seasonally changing temperature in the burial experiment.

#### Connecting the field and laboratory experiments

**Fig 13:**
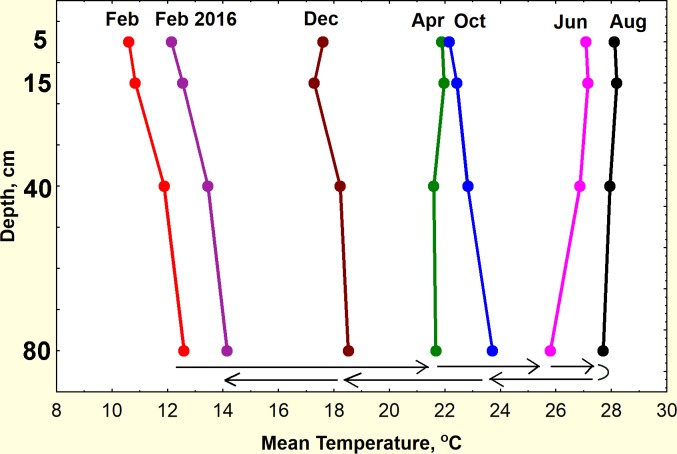
Mean soil temperatures in the burial experiment. Temperature were measured at the four burial depths during the burial periods. Although daily temperature variation differed greatly by depth, the mean temperatures were not significantly different by depth, but increased until August, and then decreased until the end of the year. The mean temperatures to which seeds were exposed at the different depths were thus similar.

The results for buried seeds and the laboratory seeds are comparable because the seeds were all sampled from the large batch of 2014 seeds. Connecting the results of the burial experiment with the simultaneous and parallel laboratory temperature experiment requires that we know the soil temperatures to which the seeds were exposed during burial. These had been recorded by the i-buttons in the buried dishes. Daily temperature *variation* decreased greatly with depth, but the mean temperature did not, although of course, it changed with the seasons (Fig 13). Thus, burial at different depths exposed seeds to non-significant differences in mean temperature, but large differences in daily temperature variation, and possibly soil moisture. Assuming that the average temperature is a meaningful measure of temperature's effect, then buried seeds at all depths were exposed to 11.5°C in February, 21.8°C in April, 26.8°C in June, 28°C in August, 22.8 in October, 17.9 in December and 13.1 in February 2016. However, the mean temperature in April, a period of rapid warming, was probably lower—because of technical failures, only the last half of April was recorded. Extrapolation between early March and mid-April suggested that mean soil temperature was about 18°C at the time the seeds were buried. This was confirmed by the record from March 2013, when mean soil temperature increased 0.2°C per day, a rate that would give an early April 2015 temperature of about 18°C. By late April, the temperature was 21.8°C.

Can the laboratory results account for the burial results? Mean soil temperatures increased from February to August (Fig 12A, right scale), and decreased from August to February 2016, so that there is some level of equivalence in the x-variables of both the laboratory and field experiments, in the sense that date corresponds to a mean soil temperature. Thus, just as germination rates peaked at 15°C for sizes 14 and 18, so they also peaked in April in the burial experiment as the soil temperature passed from 18 to about 22°C. Thus, soil temperatures were too low for rapid germination in February and too high by August, resulting in the April peak and the August minimum. As the soil cooled again after August, germination peaked as the temperature passed through 18°C in December, and then slowed again as it fell to 13°C in February 2016. These patterns can be largely accounted for by the temperature exposure during burial. The lack of a main effect of depth of burial and its small interactions also support the importance of temperature, as mean temperature varied so little with depth (Fig 13).

Size 12 and 20 seeds were less sensitive to temperature, the former simply unaffected by temperature except at 10°C, and the latter decreasing steadily with increasing temperature (Fig 12B). Because the germination rate of both sizes in the burial experiment gradually increased during the year, and bore no relationship to the changes in temperature, it suggested that germination was driven simply by elapsed time.

#### Daily progress of germination

**Fig 14:**
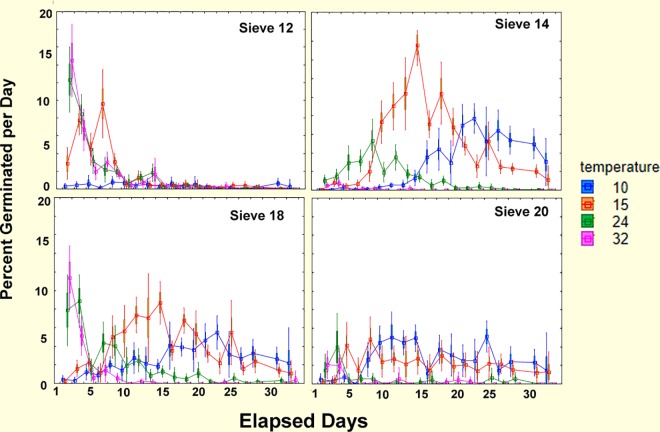
The daily progress of germination in the laboratory experiment, shown as percent germinating by elapsed day (n = 5). The timing of germination differed for seeds of different sizes. The largest seeds (size 12) mostly germinated within less than 10 days, size 14 mostly germinated after a lag, but did not germinate at 32°C. Size 18 seeds showed a mixed pattern with the two higher temperatures inducing rapid germination, and the lower two slower and more prolonged germination. The smallest seeds (size 20) mostly germinated at low rates throughout the period at the two lower temperatures, but showed little germination at the two higher temperatures.

Inspection of the day by day rates of germination in the laboratory, Feb.-Aug. (Fig 14) revealed differences in the rapidity of response to temperature. The largest seeds germinated very quickly, with midsized seeds germinating more slowly but peaking by the middle of the test period, except for the late peak of size 14 seeds at 10°C. Such rapid germination suggested that the seeds were responding to the earlier, lower April soil temperatures. However, in the laboratory as well as in the field, size 20 seeds were relatively insensitive to both month of burial and temperatures.

#### Overview of germination

Generally then, the patterns of germination after burial result from optimal germination of seeds of different sizes (and species) at different temperatures. The effect of the date of burial was therefore largely an effect of mean soil temperature, which increased until August, and decreased thereafter. Different species responded differently to date and temperature, resulting in more complex interactions, but overall, time and temperature account for most of the germination. Thus, the mixture of species and their proportions suggest that the ants can count on some germination throughout the year, regardless of the depth of storage chambers.

#### 2015 seeds, laboratory germination

A second set of seeds was collected from several harvester ant nests in September of 2015. In October and December, these were set up for laboratory germination tests identical to those for the 2014 seeds. Thus, at the time of testing, these seeds probably contained a substantial number of seeds collected by ants in 2015. Month of testing and seed size had large main effects (respectively, F_1,128_ = 42.1; p<0.00001; F_1,128_ = 18.4; p<0.00001). Germination increased from 7% to 10% from October to December, and germination of size 18 and 20 seeds was about three times as high as size 12 and 14. This was because the germination of the larger seeds increased much more strongly from October to December (month by size interaction: F_1,128_ = 11.2; p<0.00001). The temperature by size interaction was also significant (F_1,128_ = 13.2; p<0.00001) with each size responding differently to increasing temperature (except for size 12, which did not respond).

In general, the germination rates of the 2015 seeds, like those of the 2014 seeds, changed with month, temperature, seed size and their various interactions. However, overall germination was lower for the 2015 seeds, with a maximum of about 35%, about half the maximum rate for 2014 seeds. The 2015 seeds were tested in the same year they were collected, while the 2014 seeds were tested in the following year. It is likely that each batch of seeds has a unique history in the sense that there is variation in when each seed was produced, when it was collected and what species it is. It is therefore unlikely that the germination patterns of different batches of seeds will be identical.

### Do seeds germinate in *P*. *badius* storage chambers?

Having established that seeds do indeed germinate when buried in chamber-like containers (porous Petri dishes), the question remains whether they do so in natural chambers within the ants' nests. If seeds do indeed sometimes germinate during underground storage, and are quickly used by the ants, then preventing ant access to part of a seed chamber should allow germinating seeds to accumulate there. We dug a pit adjacent to active colonies and carefully dug laterally at a depth between about 30 and 90 cm until the edge of a seed chamber was exposed. A strip of sheet metal was then bent into a semi-circle and carefully pushed into the sand so that it separated the seed chamber into two parts, one to which the ants had access and one to which they did not. The pit was then filled in. After two to three weeks, the pit was again excavated, the divided chamber located, and the seeds on the access and no-access sides collected separately. Germination was assessed in the laboratory under a dissecting microscope.

**Fig 15:**
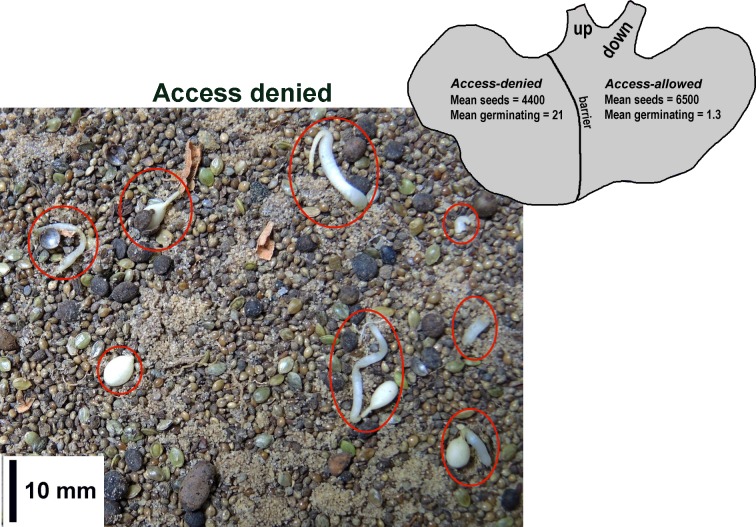
Chamber division experiment. When subterranean seed-storage chambers were divided with metals strips such that the ants had no access to one side, more germinating seeds were found on the access-denied side. Germinating seeds are circled in red. In this example, most are large.

Between December 2015 and April 2016, a total of nine replicates were successful, in the sense that the ants did not breach the barrier dividing the chamber, and seeds could be collected from both sides of the barrier. A mean of 4400 seeds were recovered from the access-denied sides, and 6500 from the access-allowed. The mean of the number of germinating seeds on the access-denied side was 21 (s.e. 7.3) and on the access-allowed side only 1.33 (s.e. 0.6) (Fig 15). A Chi-square test showed these differences to be highly significant (χ^2^ = 236.6; df = 17; p < 0.000001), confirming that germinated seeds are much more likely to occur on the side to which the ants do not have access. It follows that the rarity of germinating seeds on the access-allowed side resulted from the removal of germinating seeds by the ants, most likely to feed to the larvae, as in the experiments above.

### Species identity of the seeds

**Fig 16:**
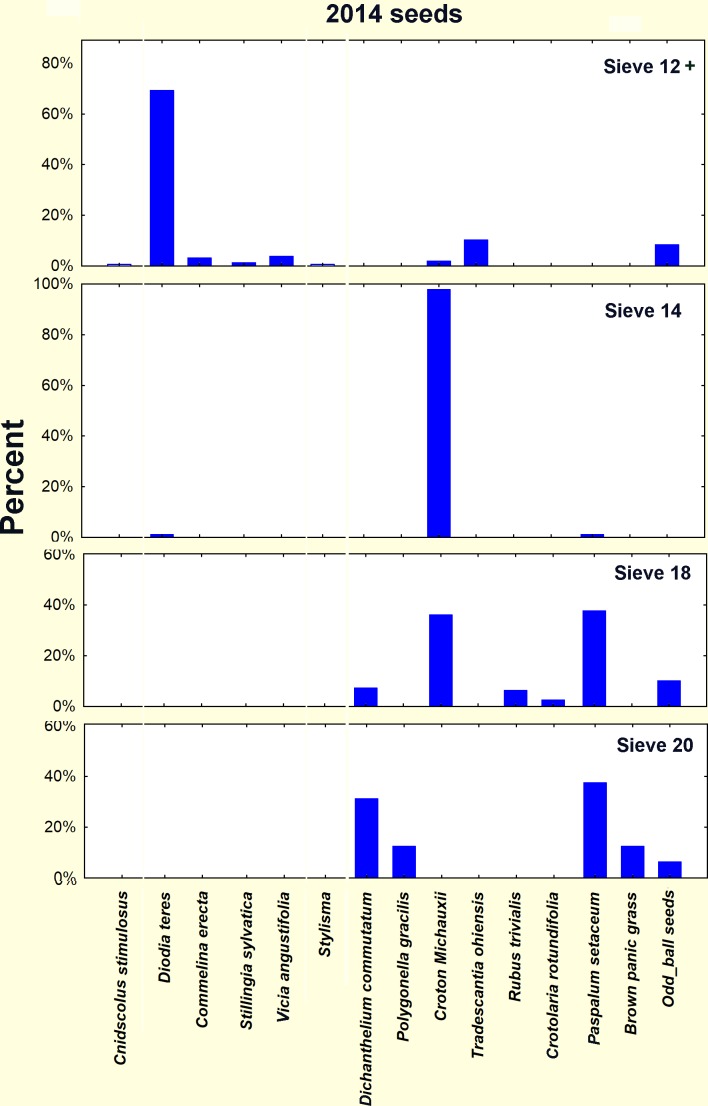
Composition by species of the 2014 seeds. The size classes differed in the degree to which they were dominated by one or two species. Size 14 consisted of almost entirely *Croton michauxi*, which was also abundant in size 18 seeds. Size 18 and 20 had substantial species diversity, and size 12, although diverse, was strongly dominated by *Diodia teres*.

The seed size classes based on retention on sieves were composed of different species (Fig 16), and their differences in germination patterns were largely differences in species composition. In the 2014 seeds, each size class was dominated by one to a few species (Fig 16). Given the differences in composition, it can be reasonably said that the temperature-indifferent (except at 10°C) germination rate of the size 12 group was largely that of *Diodia teres* (poorjoe) The patterns of the size 14 were entirely that of *Croton michauxi* (Michaux's croton, or rushfoil) because these were 98% of the seeds in this class. The similarity of germination patterns between sizes 14 and 18 was probably because size 18 shared a substantial proportion of *Croton michauxi*. Moreover, the higher April peak of size 14 than 18 was likely due to the higher proportion of *C*. *michauxi*. The smallest seeds (size 20) were largely composed of three species of grass, along with *Polygonella gracilis* (tall jointweed), so that the gradual decrease in germination with increasing temperature may be the differential responses of the several species. Of course, from the ants' point of view, the feature of importance is the seasonal germination pattern, not the species composition.

The 2015 seeds differed somewhat in composition from the 2014 seeds. In 2015, size 12 contained a substantial amount of *Rhus copalina* (sumac) in addition to *Diodia teres*. Size 14 was almost exclusively *Croton michauxi* as in 2014. In contrast to the multiple species in sizes 18 and 20 in 2014, in 2015 size 18 consisted of mostly of the grass *Paspalum setaceum*, and size 20 of the grass *Dicanthelium commutatum*.

### Seeds then and now

**Fig 17:**
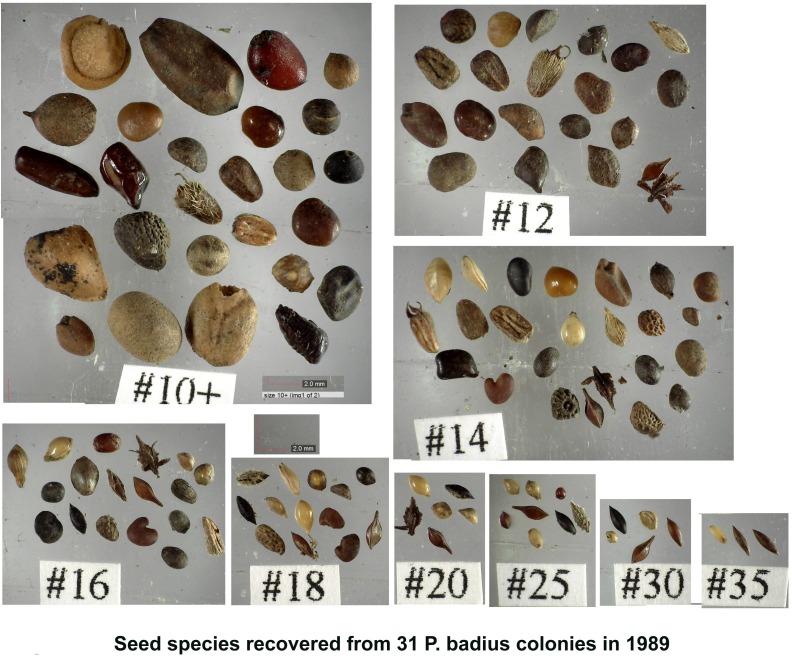
The species of seeds recovered from 31 *P*. *badius* nests in 1989 [42]. The seeds were sifted by size, and the sieve on which they were retained is indicated under each image. The approximately 50 species of seeds will be identified in another publication. Several species varied in size and were retained on more than one sieve.

All the seeds collected by WRT during the excavation of 31 *P*. *badius* colonies at Ant Heaven in 1989 [19,42] had been stored and were available for analysis. A comparison of the patterns of storage and the species of seeds available in 1989 and 2014–15 seemed of interest. Fig 17 shows the species of seeds present in the 1989 samples (the species identification of each will be published in a separate paper).

**Fig 18:**
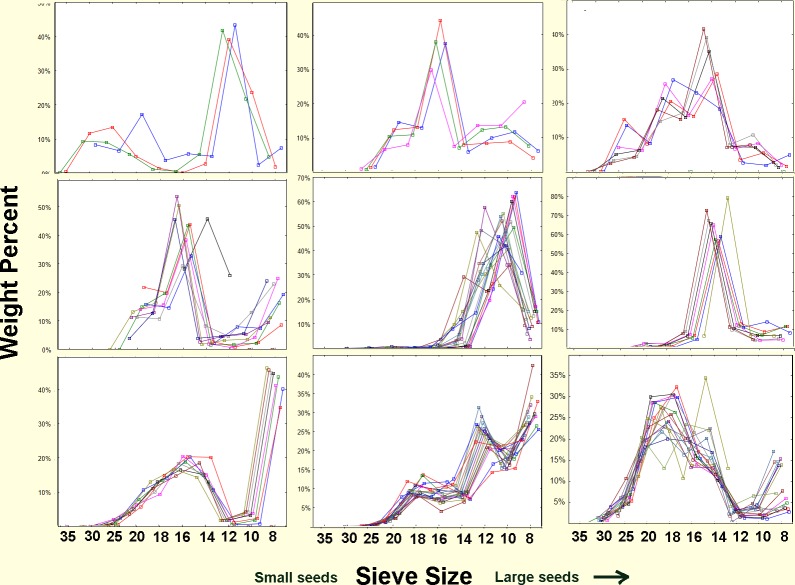
Seed size distributions among chambers and nests. Nine representative colonies out of the 31 in the 1989 samples are shown. Each line represents seed size (weight percent) distribution in a chamber, and each panel represents a colony. Size distributions differed strongly among colonies, but varied little among chambers within each colony. The colonies shown here were selected to illustrate the variety of patterns.

Fig 18 shows the distribution of seed sizes among chambers within nine representative colonies and between them. The similarity of seed size distribution among chambers within a colony, along with the consistent differences among colonies suggests that the seeds available to foragers differed spatially (Fig 18), with particular combinations of seeds predominating in some colony foraging areas and not others. This in turn suggests that the germination patterns we described above cannot be expected to be universal, or perhaps even common, as particular germination patterns will vary according to the particular mix and age of seeds. It is also possible that colonies differed in their seed preferences.

**Fig 19:**
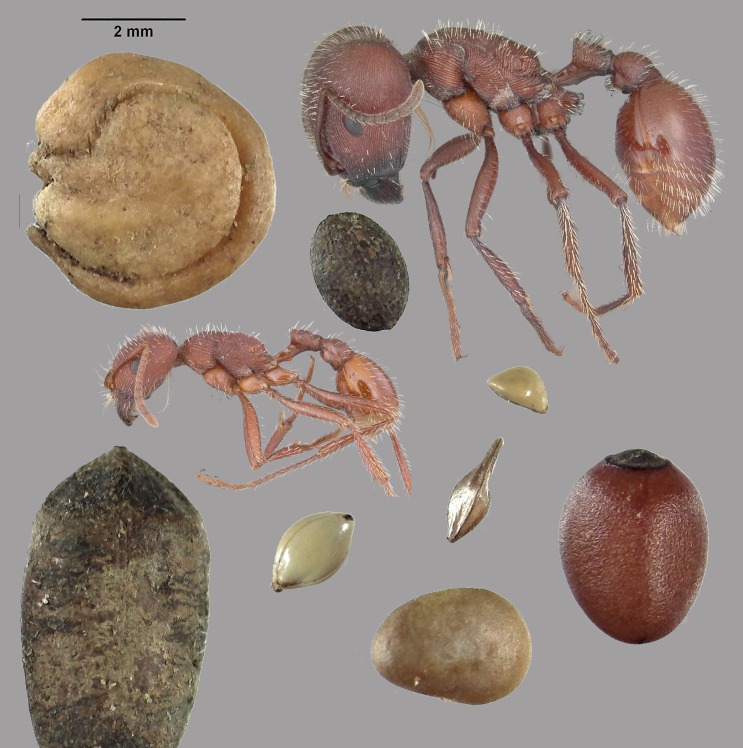
A minor and major worker of *P*. *badius* shown at the same scale as a selection of the seeds stored in the nest. In this image, the only seeds these ants can open are the three smallest sizes. Ant images modified from antweb.org. Photos by April Nobile: minor worker: URL: https://www.antweb.org/bigPicture.do?name=casent0104423&shot=p&number=1. Major worker: https://www.antweb.org/bigPicture.do?name=casent0103057&shot=p&number=1.

From the human vantage, and with unaided human vision, the size differences between the ants and many of the seeds they collect are not so obvious, but they becomes strikingly obvious when the seeds and the ants are both enlarged and shown on the ant scale (Fig 19). It is hard to imagine an ant breaching the seed coats of these larger seeds, given they must do so with their much smaller mandibles.

**Fig 20:**
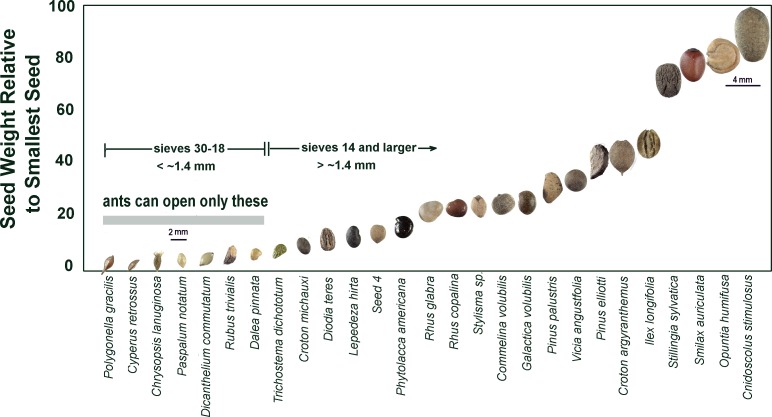
Relative size of some of the seeds found in *P*. *badius* nests. The seeds are shown in the order of their relative weight, relative to the smallest (lightest) seed, *Polygonella gracilis*. All seed species varied in weight, some substantially, so these relative weights and the order of weights is approximate.

Another way to visualize the distribution of seed sizes more vividly is to compute the relative sizes of seeds found in *P*. *badius* nests. This was done by dividing the mean weight of each species of seed by the mean weight of the smallest seeds (*Polygonella gracilis*). In Fig 20, these relative weights are plotted for 27 identified species arranged in order of their relative size. Images of the seeds at the same scale are used as data points. The largest seeds were almost 4 mm across and 80 to 90-fold as heavy as the smallest. Upon germination, such a seed would provide vastly more food than the small seeds the ants can open on their own.

**Fig 21:**
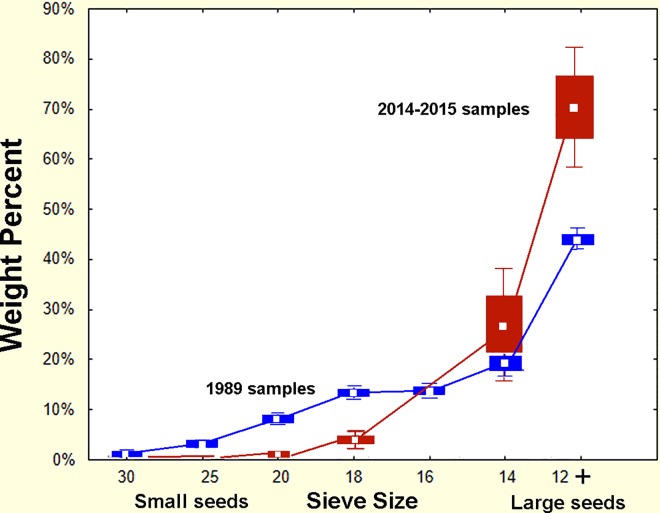
Comparison of the seed size distributions in 1989–90 with current distributions. Small seeds are currently less abundant, and large ones relatively more abundant than in 1989, probably because of successional changes in the site. The ants thus are currently able to open a smaller proportion of the seeds than in 1989, and probably depend more heavily on seed germination. (Boxes = s.e., whiskers = 95% conf. intervals).

A comparison of the overall size distributions in the 1989 samples with the 2014–15 samples shows substantial differences (Fig 21). In particular, small seeds are currently less abundant than they were in 1989. Most of the small seeds are of grasses, mostly *Dicanthelium commutatum* and *Paspalum* spp., seeds the ants can readily open. These differences are probably the result of successional changes in the plant community over this period. The current status of the site is much less grassy and later succession with larger trees than in 1989, for it had been clearcut in the mid 1970s.

## Discussion

**Fig 22:**
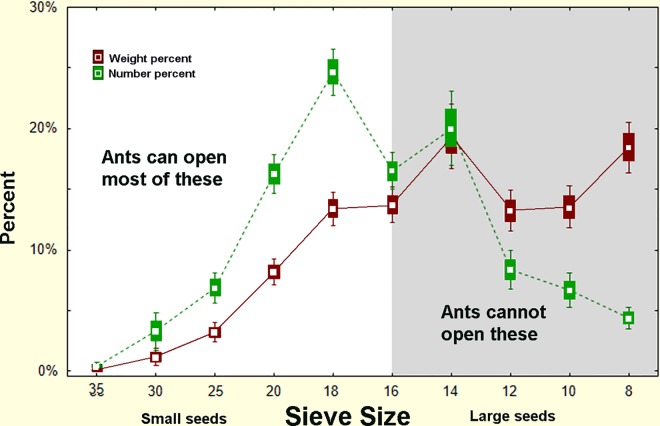
The proportion of seed resources by seed size. Weight percent of total in relation to seed size in the 31 colonies in the 1989 samples. Approximately 70% of seed biomass and 50% of seed numbers was contained in seeds that the ants cannot readily open. However, patterns differed greatly among colonies (Fig 18).

The harvester ant, *P*. *badius*, collects and stores seeds far larger than it can easily open for consumption. While the small seeds are readily opened and consumed upon demand, the large seeds are stored in damp, underground chambers where, depending on seed species, soil temperature, elapsed time and season, they germinate, thus splitting the tough husk, malting the seed and making it available as food for the ants. Looking backwards, it would be surprising if ants had not evolved this behavior, for germination would seem almost inevitable, would make the seeds more readily digestible and would greatly expand the resource base of the colonies practicing this behavior. From the ants' point of view, collecting large seeds that can be used only upon germination greatly increases the resources available to the ants—approximately 70% of the stored seed biomass, 50% by number, is in seeds the ants cannot open (Fig 22). In addition large seeds offer a greater volume of food per foraging trip, despite the delay in payoff. While individual seed choices by foragers may be related to body size of the ant [6,7,8], seed abundance [9], caloric value [10,11,12], toxicity [13] and novelty[11], colonies (rather than foragers) may favor the accumulation of a variety of seeds because of staggered germination times.

Foragers collect all sizes of seeds, but the seeds accumulate in subterranean storage chambers at size-specific rates that result from the rate of collection minus the rate of use. Because the ants open and consume the smaller sizes at a high rate, these accumulate slowly or not at all. In contrast, because the ants depend on germination (a low rate) to consume the larger ones, these accumulate to compose a large proportion of the stores. It seems possible that accumulation of large seeds occurs over the life of the colony, for colonies move their entire seed stores when they relocate into new nests [43]. When small seeds are abundant, the seed stores may contain a substantial proportion of them, but when they are uncommon, the stores may consist of almost only large seeds. The highly variable size distribution of the 1989 samples probably resulted from spatial variation of the seed-producing plants, and the difference between 1989 and 2014 suggests that temporal changes in vegetation at the same site may also play a role.

Major workers extend the range of seed sizes the ants can open themselves, but not by much, calling into question the suggestion that majors are a seed-milling caste [44,45].The largest seeds that the ants can readily open are 1 mm wide (size 18) and average about 0.8 mg in weight. A size 10 seed (2 mm wide) cannot be opened by the ants, but averages about 7.3 mg, thus containing about nine times as much nutrition as the size 18 seed. For size 25 vs. size 10 seeds, this ratio is about 15, certainly worth the wait. The payoff would be approximately the same if the large seed germinated in the time it took to open and consume 15 small ones.

Seeds are clearly broken into chunks and fed to larvae [32,35], but adult workers also ingest seeds, as evidenced by the presence of dyed seed chunks in worker crops and midguts when colonies were fed dyed seeds (Nichole Ramirez, unpublished study, with permission). It is unclear whether workers eat seeds during the winter when they do not forage-—the ratio of stored seeds to worker number or weight does not change during the winter months [18], but it is possible that the rate of use is too low to be detectable over inter-colony variation, or that worker mortality keeps pace with seed use. It is also possible that workers pass the winter on stored fat and do not feed at all [42]. The unchanging ratio of seeds to workers in *P*. *badius* is in contrast with that in *P*. *rugosus* and *P*. *subnitidus*[16]. MacKay [20] argued that seed storage is neither an adaptation for bad years, nor for overwintering, but protects against predation because it allows the nests to be closed for long periods.

It is interesting that in spite of the rather substantial rates of germination in our burial and laboratory experiments, germinating seeds are rather uncommon in excavated seed stores (although Wheeler [33] reported abundant germination in winter months), probably because the ants find and consume them quickly, as suggested by the chamber-division experiment. It is also possible that packed as densely as they are, the seeds mutually inhibit germination to some degree [28], a possibility that remains to be tested.

Our experiments used seeds separated by size, but the germination patterns are almost certainly characteristics of the species, not the seed size. It seems likely that each species germinates at rates determined by particular combinations of season, temperature, moisture and seed age. We tested only a small number of batches of seeds taken from a few harvester ant nests. Our results should be seen as an example of what may be available to the ants. Actual availability for each particular colony will depend upon the mix of seed species collected, elapsed time since collection, the season, soil temperature and soil moisture (which we did not test). These complexities suggest that the lives and success of harvester ant colonies involve many gambles, some of them choices made by the ants and some simply the capriciousness of their world.

If the germination rates in our burial and laboratory experiments are similar to those in the storage chambers of natural nests, then considerable turnover of seeds must occur over the annual cycle. Large and medium seeds would turn over mostly in the spring and fall, driven by optimal soil temperatures and germination rates between 50% and 80%. Very large and small seeds, with their germination rates of 15% to 30%, would turn over at a gradually increasing rate from spring to winter (Fig 12A), possibly in relation to some aging process or elapsed time. Whether such rates of turnover actually occur remains to be tested. Also, it should be noted that these patterns will depend upon the particular combinations of seed species, including species not in the samples we used.

Storage of wild seeds for germination is possible because in contrast to domestic seeds, wild seeds do not germinate immediately upon burial in moist soil. Rather, they form a soil seed bank from which seeds are withdrawn (germinate) only gradually and under particular conditions, thus assuring germination under favorable seasonal and environmental conditions, and hedging against bad gambles. The ants probably do not manipulate these conditions, but merely gamble that the gradual germination of a variety of species will provide them with a continuous supply of food. Interestingly, when colonies of *P*. *badius* were experimentally prevented from foraging, they did not access their seed stores, reducing larval survival, alate production and worker fat stores [13,15]. This outcome may have been the result of insufficient rates of seed germination within their seed stores once the income of small seeds was experimentally curtailed.

Several 19th and early 20th century observers noted germinating seeds in harvester ant nests, and seemed to accept that the ants might actually be using these [32,33]. The wide variation in their observations are not surprising considering that the ant species, seed species, season, continents and even centuries were different. Nevertheless, since these early observations, the implicit assumption of most studies has been that the ants are able directly to use the seeds they collect, and that the seeds are mostly prevented from germinating. Thus, most recent studies have focused on seed collection and related choices, rather than on seed consumption. Our studies have shown that one harvesting ant species in one location exploits germinating seeds. The difference in the species composition of chaff in at least one other location suggests that this phenomenon is widespread in *P*. *badius*. It remains to be seen whether other harvesting ant species also exploit seed germination to their benefit. Such exploitation seems more likely for species in moister habitats, but it is also possible that it occurs in more arid zone species if their stores are maintained in moist soil.

The question that remains is whether or not the exploitation of germinating seeds should be considered alongside other farming practices in ants. *P*. *badius* workers collect seeds too large to be opened with their own mandibles, excavate a nest for storage and store the seeds in moist chambers with specific temperatures, all of which promote germination in the same way that planting a seed might. This action may yet be too passive to be termed farming, but just as fungus gardening ants forage for items to feed fungus and herdsman ants move honeydew producing insects to different plants [1], seed harvesting ants change external conditions to promote growth in another living thing for their own benefit.

## Supporting Information

S1 Table Seed size data by colony for the 1989 seeds. (XLSX)

S2 Table Germination data for the burial experiment. (XLSX)

S3 Table Germination data for the laboratory temperature experiment. (XLSX)

S4 Table Analysis of variance tables for burial and laboratory experiments. (DOCX)
