# Supplementary material for: The Florida Harvester Ant, Pogonomyrmex badius, Relies on Germination to Consume Large Seeds
Source: PLoS One. 2016 Nov 28;11(11):e0166907. doi: 10.1371/journal.pone.0166907 (PMC5125654; doi:10.1371/journal.pone.0166907)
Supplement: S4 Table — (DOCX) [file pone.0166907.s004.docx]

**Analysis of variance tables**

**Table 1**. Analysis of variance of the burial experiment: Proportions were transformed to arcsin square root for analysis. Significant effect are in bold.

| Effect | Univariate Tests of Significance for arcsin % germinated Sigma-restricted parameterization Effective hypothesis decomposition. 2014 seeds, run Jan.-Dec. 2015 | | | | | |
| --- | --- | --- | --- | --- | --- | --- |
|  | \| SS \| \| --- \| | \| % of factor SS* \| \| --- \| | \| Degr. of Freedom \| \| --- \| | \| MS \| \| --- \| | \| F \| \| --- \| | \| p \| \| --- \| |
| \| Intercept \| \| --- \| | 110.55 |  | 1 | 110.55 | **8812.2** | **0.000000** |
| \| Date of check (season) \| \| --- \| | 7.46 | 26% | 5 | 1.49 | **118.9** | **0.000000** |
| \| Depth \| \| --- \| | 0.08 | 0% | 3 | 0.03 | 2.1 | 0.103363 |
| \| Sieve size \| \| --- \| | 10.08 | 35% | 3 | 3.36 | **267.9** | **0.000000** |
| \| Date of check*Depth \| \| --- \| | 0.57 | 2% | 15 | 0.04 | **3.0** | **0.000141** |
| \| Date of check*Sieve size \| \| --- \| | 9.00 | 32% | 15 | 0.60 | **47.8** | **0.000000** |
| \| Depth*Sieve size \| \| --- \| | 0.25 | 1% | 9 | 0.03 | **2.3** | **0.018443** |
| \| Date of check*Depth*Sieve size \| \| --- \| | 0.98 | 3% | 45 | 0.02 | **1.7** | **0.003265** |
| \| Error \| \| --- \| | 4.60 |  | 367 | 0.01 |  |  |

*Date, sieve size and date x sieve account for 93% of the factor-associated variance.

**Table 2**. Analysis of variance of the proportion of seeds germinating in the laboratory experiment. Proportions were arcsin square root transformed for analysis. Significant effects are in bold. "Sanple" in this experiment is the parallel of "date of burial" in the burial experiment.

| Effect | Univariate Tests of Significance for arcsine % germinated. Sigma-restricted parameterization Effective hypothesis decomposition .2014 seeds, run Jan.-Dec. 2015 | | | | | |
| --- | --- | --- | --- | --- | --- | --- |
|  | \| SS \| \| --- \| | \| % of factor SS* \| \| --- \| | \| Degr. of Freedom \| \| --- \| | \| MS \| \| --- \| | \| F \| \| --- \| | \| p \| \| --- \| |
| \| Intercept \| \| --- \| | 106.94 |  | 1 | 106.94 | **13123.1** | **0.000000** |
| \| sample \| \| --- \| | 0.38 | 2% | 5 | 0.08 | **9.3** | **0.000000** |
| \| temperature \| \| --- \| | 6.36 | 28% | 3 | 2.12 | **260.3** | **0.000000** |
| \| sieve size \| \| --- \| | 1.12 | 5% | 3 | 0.37 | **45.9** | **0.000000** |
| \| sample*temperature \| \| --- \| | 1.96 | 9% | 15 | 0.13 | **16.1** | **0.000000** |
| \| sample*sieve size \| \| --- \| | 1.86 | 8% | 15 | 0.12 | **15.2** | **0.000000** |
| \| temperature*sieve size \| \| --- \| | 8.67 | 38% | 9 | 0.96 | **118.2** | **0.000000** |
| \| sample*temperature*sieve \| \| --- \| | 2.25 | 10% | 45 | 0.05 | **6.1** | **0.000000** |
| \| Error \| \| --- \| | 3.00 |  | 368 | 0.01 |  |  |

*Temperature, and temperature x sieve size explain 66% of the variance associated with the factors.
